# Persistent Menorrhagia and Hemorrhagic Ovarian Cyst in a Patient With Bernard-Soulier Syndrome: A Case Report

**DOI:** 10.7759/cureus.76233

**Published:** 2024-12-22

**Authors:** Maria Hasani, Sadia Rounak Shriya, Mariyam Thahira, Tasnima Tayb, Hasan Aal Yaseen

**Affiliations:** 1 Internal Medicine, Mohammed Bin Rashid University of Medicine and Health Sciences, Dubai Health, Dubai, ARE; 2 Hematology, Dubai Hospital, Dubai Health, Dubai, ARE

**Keywords:** bernard-soulier syndrome, heavy menstrual bleeding, hemorrhagic ovarian cyst, hmb, menorrhagia

## Abstract

Bernard-Soulier syndrome (BSS) is a rare qualitative condition of platelets wherein deficiency of platelet surface glycoproteins (GP) Ib, IX, and V forms the Ib-IX-V complex, leading to impaired hemostasis. Although it commonly presents as prolonged bleeding in general, women in the reproductive phase report additional complications during menstruation, pregnancy, and childbirth. In women of reproductive age, menorrhagia is a frequent complaint. It is reported that secondary to hormonal and other causes, hemostatic disorders are present in a substantial proportion of women presenting with persistent menorrhagia. Women with bleeding disorders also hold an additional risk of developing complications like hemorrhagic corpus luteum. While corpus luteum rupture may go unnoticed in healthy women, it can cause life-threatening intraperitoneal hemorrhage in women with bleeding disorders.

This report presents a case of a young woman with BSS who developed menorrhagia against the backdrop of a hemorrhagic ovarian cyst. Owing to the rarity of reported management of hemorrhagic cysts with underlying BSS, this report aimed to potentially serve as a guide in facilitating decision-making for physicians.

## Introduction

Bernard-Soulier syndrome (BSS) is often manifested as spontaneous and profuse bleeding. A hereditary condition usually transmitted through an autosomal recessive pattern and has been found to run in consanguineous families. In general, BSS often presents with prolonged bleeding time, enlarged platelets, and thrombocytopenia. Severe bleeding usually presents due to traumatic experiences such as surgical procedures, dental extractions, or accidents [1].

Although BSS is found in men and women, women experience additional complications particularly during the reproductive phase, owing to menstruation, pregnancy, and childbirth [2]. It is reported that hemostatic disorders can also contribute to menorrhagia. Women with bleeding disorders also develop complications related to ovarian cysts. Ovarian cysts can be functional or non-functional. Functional cysts, such as follicular cysts and corpus luteum cysts, can occur due to the menstrual cycle, while non-functional cysts include chocolate cysts (endometriotic), dermoid cysts, cystadenomas, and malignant cysts.

Ovarian cyst rupture and hemorrhage often occur in association with functional ovarian cysts. They are generally self-limiting but can develop complications. One possibility is the development of a hemorrhagic corpus luteum (HCL) due to bleeding into the corpus luteum. Initially, blood may fill the central cavity which could be self-limiting. However, it can progressively enlarge and eventually rupture. This could result in hemoperitoneum which can be detected on imaging or life-threatening hemorrhage in women with underlying bleeding disorders. Management can range from a conservative approach to laparoscopic surgery based on the severity of the presentation [3]. Management could be more challenging in such patients and must account for any additional risks posed by the underlying bleeding tendency and potential recurrence rates. This may require a multi-disciplinary approach [4].

A case of a young woman with BSS who presented with menorrhagia and hemorrhagic ovarian cyst is discussed here. Considering the limited availability of data and guidelines on managing such conditions with underlying BSS, this could potentially serve as a guide in facilitating decision-making for physicians encountering such patients in the future.

## Case presentation

A 22-year-old nulliparous Pakistani female with BSS presented with a two-day history of continuous left flank abdominal pain radiating to the back which was associated with nausea. Her last menstrual period was 22 days ago. She was previously admitted five months back due to intraperitoneal bleeding and a computed tomography (CT) pelvis during that visit had confirmed a 4.6 × 5.2 × 5.2 cm complex multi-loculated heterogeneous mass in the left adnexal region. In the subsequent follow-up visits prior to the current presentation, the size of the cyst had remained stable.

The patient was diagnosed with BSS at the age of eight months following an episode of uncontrolled bleeding of the lips. She has a history of frequent gum bleeding, epistaxis, and bruising. Her periods started at the age of 11 years and she has a history of persistent menorrhagia and iron deficiency anemia which was managed with tranexamic acid (TXA), iron, and desmopressin. There had been gradual worsening of menorrhagia and thrombocytopenia after that and the patient has received multiple platelet transfusions since then.

**Figure 1:**
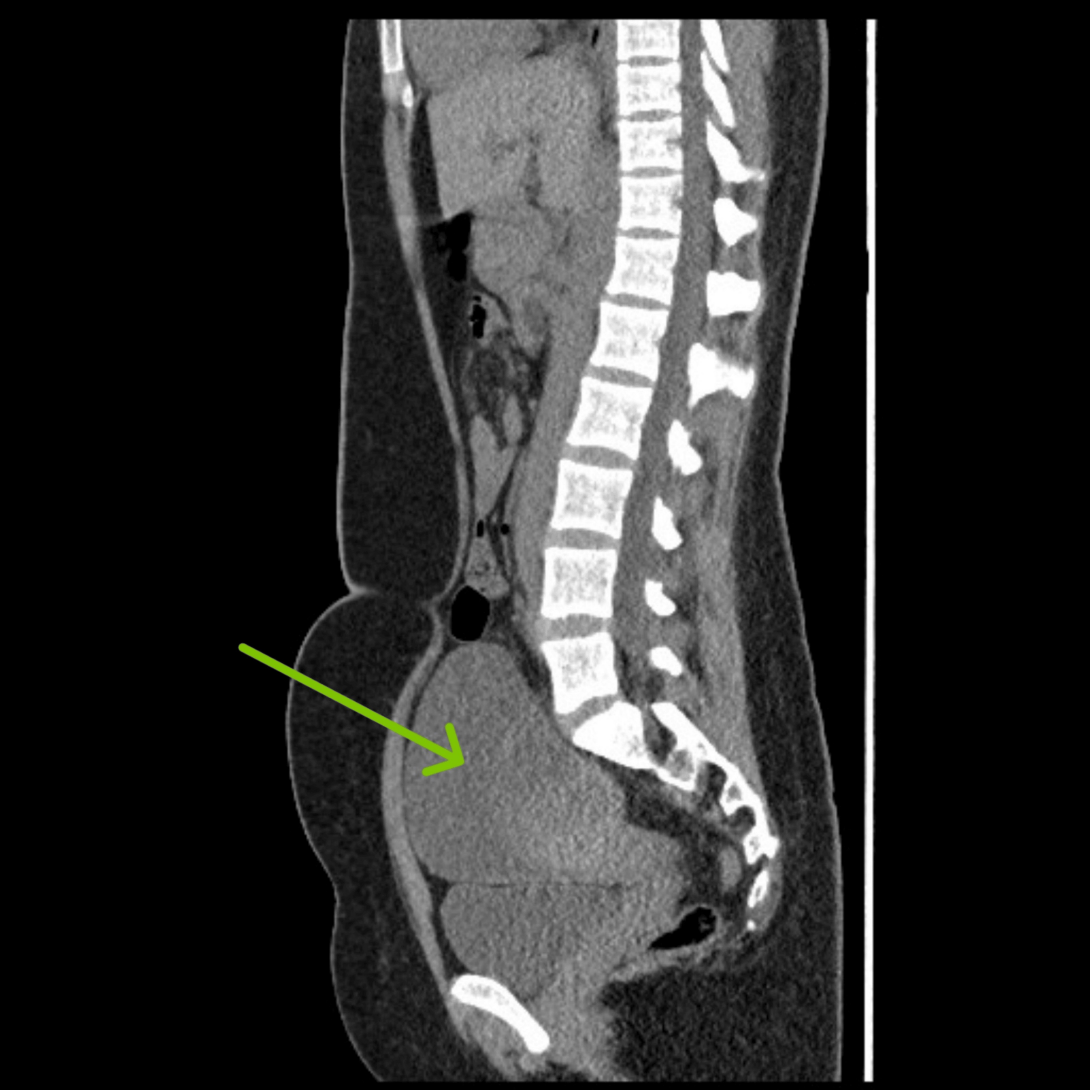
CT abdomen and pelvis, sagittal view. The arrow points to the enormous left adnexal cyst.

**Figure 2:**
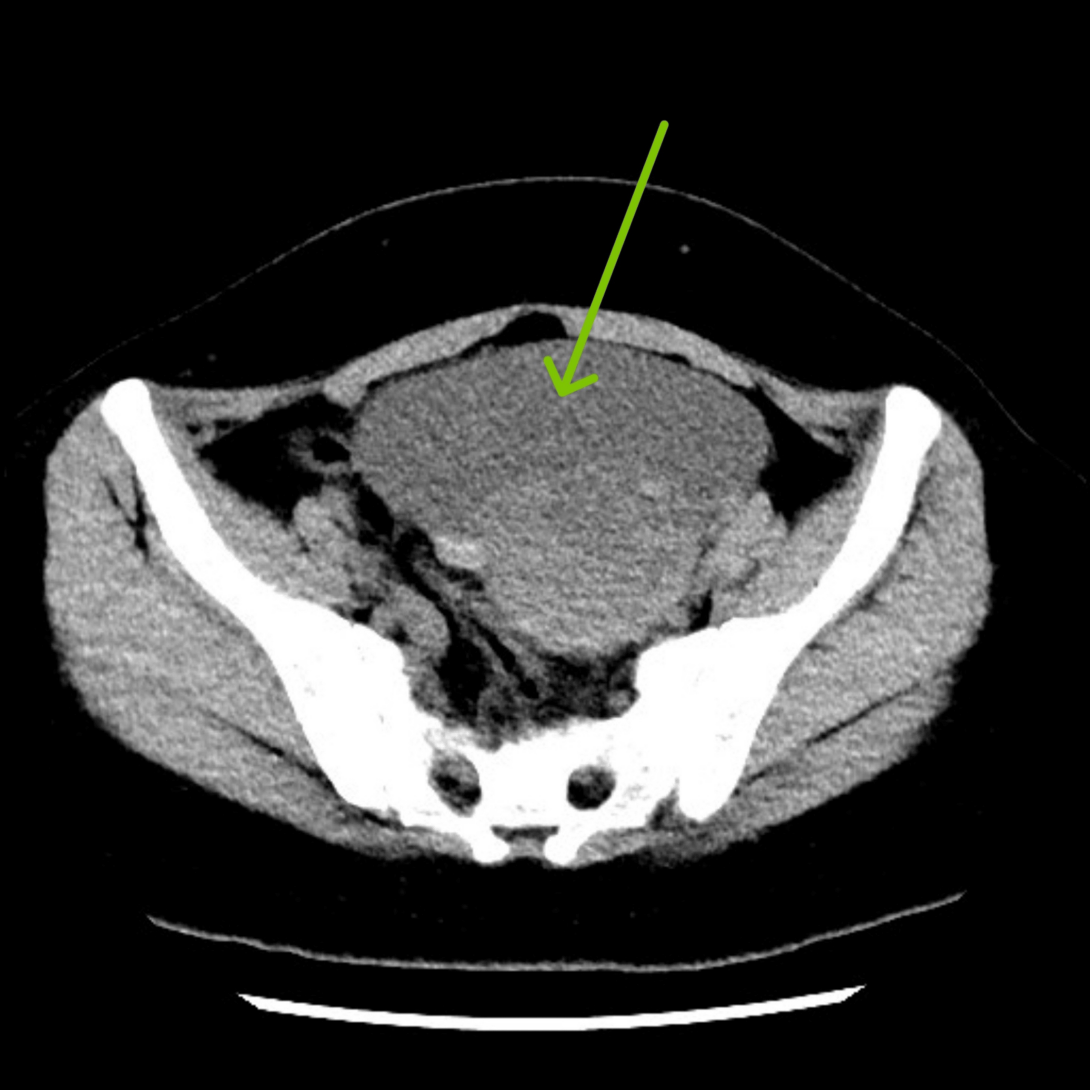
CT abdomen and pelvis, axial view. The arrow points to the left adnexal cyst in the axial view.

On current presentation, the patient denied any fever, vomiting, or other gastrointestinal and urinary complaints. Her vitals were within normal ranges. On abdominal examination, there was tenderness in the left upper and lower quadrants, which was 7/10 in severity on the pain scale, along with left costovertebral angle tenderness. No guarding or rebound tenderness was noted. The initial laboratory workup revealed thrombocytopenia with a platelet count of 17 x 10^3^/µL (range: 150-410 x 10^3^/µL), red blood cell count of 4.06 x 10^6^/µL (range: 3.80-4.80 x 10^6^/µL), hemoglobin of 12.0 g/dL (range: 12.0-15.0 g/dL), and hematocrit of 37.2% (range: 36.0-46.0%). Prothrombin time (PT) was 11.5 s (range: 9.7-11.4 s), international normalized ratio (INR) was 1.11 (range: 0.8-1.1), and activated partial thromboplastin time (aPTT) was 27.7 s (range: 27-40 s). Computed tomography (CT) of the pelvis revealed a left adnexal cyst measuring 12.1 × 10.9 cm in size with a hemorrhagic component (Figures 1, 2).

The patient had an acute on chronic septate and adhesive hemorrhagic ovarian cyst. Conservative management was tried since her initial presentation five months prior, but she presented acutely again with a progressively enlarging hemorrhagic cyst. Since there was a significant risk of ovarian rupture and intraperitoneal bleeding with potential hypovolemic shock, the decision for a cystectomy was taken. Preoperatively, her laboratory workup results were abnormal, and she received a single-donor platelet transfusion and tranexamic acid (TXA), with activated factor VIIa kept on standby (Table 1). Subsequently, she underwent a laparoscopic ovarian cystectomy.

**Table 1:** Initial laboratory results on the day of presentation. WBC: white blood cells; RBC: red blood cells; PT: prothrombin time; INR: international normalized ratio; aPTT: activated partial thromboplastin time

Parameter	Result	Reference range
WBC count	12.3 x 10^3^/µL	3.6-11.0 x 10^3^/µL
RBC count	2.67 x 10^6^/µL	3.80-4.80 x 10^6^/µL
Hemoglobin	7.9 g/dL	12-15 g/dL
Hematocrit	24.2%	36.0-46.0%
Platelets count	20 x 10^3^/µL	150-410 x 10^3^/µL
PT (next day of surgery)	12.4 s	9.7-11.4 s
INR (next day of surgery)	1.21	0.8-1.1
aPTT (next day of surgery)	28 s	27-40 s

The hemorrhagic cyst was excised*.* It had thick double walls and multiple loculations filled with blood and blood clots deemed to be due to previous multiple attacks of hemorrhage. It was firmly adherent to the left side of the uterus, bowels, and to the left pelvic wall. While removing the adhesions, the cyst was punctured and blood clots were suctioned. The part of the cyst attached to the pelvic wall was left undisturbed to avoid injury to the bowels, pelvic vessels, and ureter.

There was no active bleeding at the end of the operation and the patient tolerated the surgery well. Total blood loss was 800 mL and four units of platelets were transfused intraoperatively. Postoperatively, two units of platelets and four units of packed RBCs were transfused. Intravenous (IV) antibiotics were given for 72 hours and intravenous TXA was administered. Upon stabilization, the patient was discharged on oral antibiotics including cefuroxime and metronidazole as well as TXA. Postcystectomy, she has been following up in the hematology and gynecology clinic with good progress.

## Discussion

Menorrhagia is a common phenomenon that causes significant effects on the quality of life. Von Willebrand disease (VWD) is the most studied common primary bleeding disorder, with heavy menstrual bleeding (HMB) as a common clinical manifestation. Recently, bleeding disorders like VWD or BSS, single coagulation factor deficiencies, and platelet function disorders are found to be prevalent in menorrhagic patients [5]. According to a systematic review by Punt et al., which included data from four cohort studies, 25% of women with BSS (13/52) have a current or past history of heavy menstrual bleeding [3]. In a tertiary care center in India, out of 1100 cases of heavy menstrual bleeding (HMB), 104 had no pelvic pathology or hormonal disorders. Of these, 1% (1/104) had BSS, which accounts for 0.09% of the total cases [6]. Such primary hemostasis disorders are usually overlooked as a cause of menorrhagia, especially when these disorders do not present with anomalies in the PT, aPTT, and CBC results. Therefore, clinicians should always further investigate the possibilities of hematologic diseases.

BSS is suspected when a triad of variable thrombocytopenia, prolonged bleeding time, and abnormally large platelets with increased bone marrow megakaryocytes is observed. It is confirmed genotypically through molecular studies, or biochemically through an absence of response to ristocetin in platelet aggregation studies, or a decreased expression of platelet surface GPIb in flow cytometry. The differential diagnosis of BSS includes immune thrombocytopenia, VWD, May-Hegglin anomaly, Gray platelet syndrome, and Glanzmann thrombasthenia. Commonly an autosomal recessive disorder, BSS is observed more frequently in countries with a high proportion of consanguineous marriages, such as Iran, Pakistan, and Arab countries [7]. The World Federation of Hemophilia has reported 667 cases of BSS from 113 countries in 2017, with Iran ranking at the top.

Management of BSS depends on the severity of the condition. In most cases, patients have required treatment with whole-blood, packed RBC, or platelet transfusion. Moreover, antifibrinolytics such as tranexamic acid (TXA) appear to be successful in controlling bleeding from mucous membranes, and desmopressin acetate (DDAVP) has been proven to shorten bleeding time. Factor VIIa has been used in patients who do not respond to transfusion of platelets or DDAVP [8]. Unfortunately, factor VIIa may lead to an increased risk of thromboembolic events. Bone marrow transplantation from human leukocyte antigens (HLA)-compatible donors was noted to have success in BSS patients with platelet refractoriness and severe bleeding complications. Good clinical outcome was noted in a pair of BSS twins who received transplantation from their HLA-identical brother and a 28-year-old BSS female with peripheral blood stem-cell transplant from her brother showed normal platelet aggregation tests [9,10]. Though some authors recommend early bone marrow transplantation, most of the transplant reports were reserved only in cases of severe disease. It is important to carefully evaluate the need for hematopoietic stem-cell transplant in BSS patients due to significant risks of allograft [11].

Unfortunately, BSS patients can have gynecological complications of cysts that usually become hemorrhagic. Patients with rare bleeding disorders have been reported to have a higher incidence of hemorrhagic ovarian cysts, nearly 30% in a study of 210 patients [12,13]. The symptoms of hemorrhagic ovarian cysts can vary from asymptomatic to sudden onset of lower abdominal pain. The pain is due to irritation of the visceral peritoneum, blood flow into the abdomen, or stretching of the ovarian cortex. Other symptoms can include nausea, vaginal bleeding, or hemorrhagic shock in extreme situations. To note, it is often difficult to clinically differentiate between a hemorrhagic cyst and a ruptured hemorrhagic cyst. Though hemorrhagic cysts in most patients can resolve within eight weeks, further surgical evaluation is warranted if subsequent imaging shows an increase in cyst size, local compression, massive effusion, or cysts in postmenopausal women. However, acute management of hemorrhagic ovarian cysts needs to be managed medically with platelet concentrate and antifibrinolytics or through surgical therapy [11,14]. In the case of a 16-year-old known BSS patient who was admitted with signs of hemorrhagic shock and peritoneal irritation, imaging showed a ruptured ovarian cyst and large abdominal effusion. With no improvement after medical interventions, resection of the cyst, repetitive lavage, and complete liquid aspiration were performed [15].

Though there is limited reported surgical management of hemorrhagic ovarian cysts in BSS, management is usually laparoscopic, and preoperative preparations require the administration of concentrated platelets, fresh frozen plasma (FFP), or packed RBCs. Moreover, all patients must be screened for human leukocyte antigens (HLA) and platelet antibodies prior to surgery. This is important because BSS patients are prone to develop platelet antibodies due to repetitive transfusion leading to platelet transfusion refractoriness. In addition, HLA alloimmunization can also occur [16]. Preoperative antifibrinolytics can be commenced and continued for one to two weeks. Patients with bleeding disorders have a higher risk of recurrence of cysts. Surgical drainage or removal of cysts is the only known method to prevent the rupture of an existing ovarian cyst. However, the need for gonadotropin-releasing hormone (GnRH) analogs or estro-progestinics is important to prevent ovulation and thereby avoid further episodes of hemorrhagic cysts in patients with bleeding disorders [17].

## Conclusions

In cases complicated by the presence of both cysts and bleeding disorders, a high index of suspicion and care must be taken to aid early detection. Once diagnosed, management needs to be tailored according to the severity of the case and the risk of bleeding from a hemorrhagic ovarian cyst compared to the risk of bleeding due to operative interventions. Although conservative management may be sufficient to control the initial bleeding in some cases, multiple episodes may require surgical interventions, such as laparoscopic cystectomy or oophorectomy, after considering various factors.
